# Dysregulated Metabolism in EGFR-TKI Drug Resistant Non-Small-Cell Lung Cancer: A Systematic Review

**DOI:** 10.3390/metabo12070644

**Published:** 2022-07-14

**Authors:** Julia Babuta, Zoe Hall, Toby Athersuch

**Affiliations:** Biomolecular Medicine Division of Systems Medicine, Department of Metabolism, Digestion and Reproduction, Imperial College London, London SW7 2AZ, UK; zoe.hall@imperial.ac.uk (Z.H.); toby.athersuch@imperial.ac.uk (T.A.)

**Keywords:** NSCLC, EGFR-TKI, metabolomics, lipidomics, drug resistance, metabolism, metabolites

## Abstract

Drug resistance is a common barrier to continued effective treatment in cancer. In non-small-cell lung cancer (NSCLC), tyrosine kinase inhibitors that target the epidermal growth factor receptor (EGFR-TKIs) exhibit good efficacy in cancer treatment until acquired resistance occurs. It has been observed that drug resistance is accompanied by numerous molecular-level changes, including significant shifts in cellular metabolism. The purpose of this study was to critically and systematically review the published literature with respect to how metabolism differs in drug-resistant compared to drug-sensitive NSCLC. Understanding the differences between resistant and sensitive cells is vital and has the potential to allow interventions that enable the re-sensitisation of resistant cells to treatment, and consequently reinitiate the therapeutic effect of EGFR-TKIs. The main literature search was performed using relevant keywords in PubMed and Ovid (Medline) and reviewed using the Covidence platform. Of the 1331 potentially relevant literature records retrieved, 27 studies were subsequently selected for comprehensive analysis. Collectively, the literature revealed that NSCLC cell lines resistant to EGFR-TKI treatment possess characteristic metabolic and lipidomic phenotypic signatures that differentiate them from sensitive lines. Further exploration of these reported differences suggests that drug-resistant cell lines are differentially reliant on cellular energy sources and that modulation of relative energy production pathways may lead to the reversal of drug resistance.

## 1. Introduction

Lung cancer is the second most diagnosed cancer world-wide [1], with NSCLC accounting for 80% of lung cancer cases [2,3]. NSCLC is a lethal and difficult to treat cancer, with 16% morbidity and 18.4% mortality [4]. One of the most common driving mutations in NSCLC patients is found in the epidermal growth factor receptor (EGFR). Such cancers respond well to EGFR-tyrosine kinase inhibitors (EGFR-TKIs) [3], which target the ATP binding site of the TK domain of EGFR, blocking downstream signalling pathways such as Ras/MAPK and PI3K/Akt [3]. These signalling pathways are heavily involved in cell proliferation, and when constitutively activated in NSCLC patients, can allow for increased proliferation whilst simultaneously evading apoptosis [5].

Targeted therapies that include EGFR-TKIs are currently an effective standard of care treatment, with the first-generation TKIs-erlotinib and gefitinib-giving patients up to one year of progression-free survival (PFS) [6] before acquired resistance occurs. Resistance can arise in both an EGFR-dependent and/or an EGFR-independent manner. The most common form of EGFR-dependent acquired resistance arises as a point mutation within EGFR (EGFRm), at T790M residue occurring in around 60% of cases [6]. EGFR-independent resistant mechanisms occur as a consequence of mutations in other oncogenes such as c-MET [1] or by mutations downstream of EGFR that allow for the pathway to be constitutively activated once more, promoting tumorigenesis. It is because of this arising resistance to therapies that further generations of TKIs have been developed, including third-generation drug osimertinib (AZD9291). Osimertinib selectively targets both the initial EGFRm and T790M mutations whilst selecting against EGFR wild type (EGFRwt). However, similar to previous generations of TKIs, resistance to the newer generation TKIs is inevitable. Understanding the process of drug resistance acquisition for TKIs offers an opportunity to identify new adjuvant therapeutic targets that prevent or reverse drug resistance and reinstate the desired therapeutic effect.

**Figure 1:**
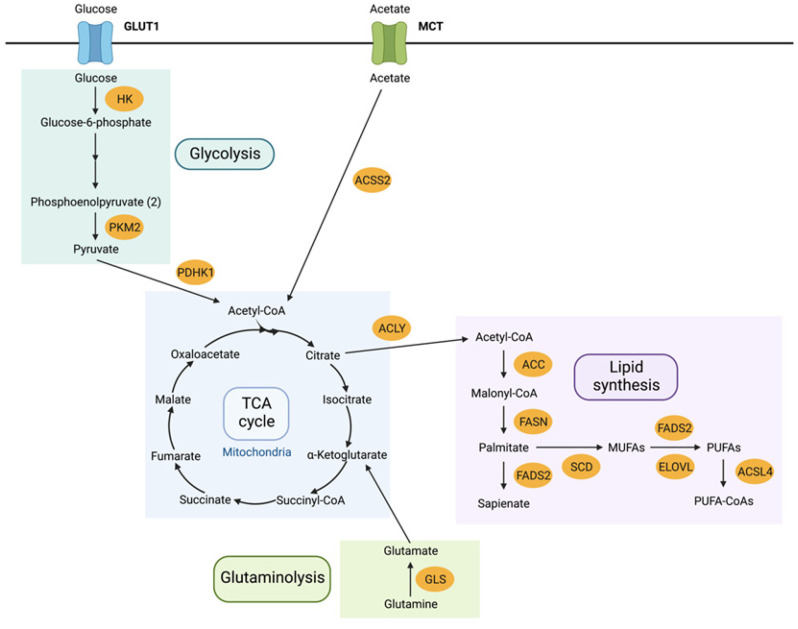
An overview of cellular metabolic key pathways; glycolysis, TCA cycle, glutaminolysis and lipid synthesis. Adapted from Koundouros et al., 2019 [13]. Abbreviations: GLUT1 – glucose transporter 1, HK—hexokinase, PKM2—pyruvate kinase M2, PDHK1—pyruvate dehydrogenase kinase 1, MCT – monocarboxylate transporter, ACSS2—acyl-CoA synthetase short chain family member 2, ACLY—ATP citrate lyase, ACC—acetyl-CoA carboxylase, FASN—fatty acid synthase, FADS2—fatty acid desaturase 2, SCD—stearoyl-CoA desaturase 1, ELOVL—elongation of very long chain fatty acids, ACSL4—acyl-CoA synthetase long chain family member 4.

The dysregulation of cellular metabolism has been acknowledged as a hallmark of cancer for over 90 years [7]. Driven by oncogenes, this phenomenon allows key steps of metabolism to be altered to favour tumorigenesis [8]. A specific example of this is the ability of cancer cells to switch their energy metabolism to favour aerobic glycolysis over oxidative phosphorylation, the key metabolic process for ATP production in healthy cells, via the mitochondria [8]. This results in an increased production of lactate from glucose even in the presence of oxygen, and is known as the Warburg effect [7,8,9]. A shift from oxidative phosphorylation can be attributed to oncogenic signalling from pathways downstream of EGFR [8]. In NSCLC specifically, EGFR signalling enhances glycolysis [10]. This phenomenon of creating additional ATP energy also promotes glucose utilisation in anabolic processes for synthesis of other vital compounds such as DNA, proteins and lipids—key building blocks required for cancer cells to proliferate [1,7,9,11]. Medes et al. discovered that tumour cells synthesize lipids from glucose or acetate, and rely upon de novo lipogenesis as the primary form of fatty acid generation [12] to allow for cancer cells’ high demand for lipids (Figure 1).

A multi-omics approach including genomics, proteomics and metabolomics allows for a wider comprehension of disease by combining information from diverse biological levels [14]. While changes at the genomic level are well documented [15], the full spectrum of metabolic contributions to drug resistance in cancer are less well defined; this review will focus on studies which measure the metabolic phenotype, including but not limited to metabolomics and lipidomics. In lung cancer, previous studies identified metabolites involved in amino acid metabolism, glycolysis, oxidative phosphorylation, gluconeogenesis and fatty acid metabolism to be dysregulated in drug-resistant cells [16].

In its broadest sense, metabolomics (commonly referred to as metabonomics, metabolic profiling, or metabolic phenotyping) is the study of measuring the levels of metabolites in biological samples such as cells, tissues and biofluids [16], allowing the metabolic state of the cell to be determined. Metabolites of interest are often intermediates of key pathways such as glycolysis, and their levels within cancer cells or in drug-resistant vs. drug-sensitive cell lines can give an indication of which metabolic pathway has been rewired [17]. Metabolomics is largely studied using liquid chromatography–mass spectrometry (LC-MS), gas chromatography–mass spectrometry (GC-MS) and proton nuclear magnetic resonance (^1^H-NMR) spectroscopy [18]. LC-MS is most frequently used due to its sensitivity and high throughput [16].

Lipidomics is the study of all the non-polar metabolites within a sample to obtain the lipidomic phenotype or lipid profile. Cancer cells heavily rely on de novo lipogenesis for energy production and membrane biosynthesis to maintain the rapid proliferation of cells [19,20,21]. In drug-resistant cancer cells, lipid metabolism, with emphasis on de novo lipogenesis, is found to be even more active [19,21]. Lipid storage and uptake of endogenous lipids is also known to increase in resistant cells [20]. De novo lipogenesis primarily occurs through the sterol regulator element binding (SREBP1) transcription factor, which activates fatty acid synthase (FASN), acetyl CoA carboxylase (ACC) and stearoyl CoA desaturase (SCD). These all play a role in fatty acid synthesis, including the synthesis of more complex lipids such as phospholipids [19,20]. Phospholipids, triglycerides and sphingolipids are examples of complex lipid species in which fatty acids are bound to a backbone and head group [13,19]. They can then be synthesised into other complex lipids, such as triacylglycerides (TAGs), diacylglycerides (DAGs), phosphoglycerides such as phosphatidic acid (PA), phosphatidylserine (PS), phosphatidylethanolamine (PE) and phosphatidylcholine (PC) [13].

Untargeted-omics studies provide a means to generate metabolomic or lipidomic profiles that are representative of the small molecule composition of samples obtained from biological systems with minimal a priori selection of analytes, but often with a substantial requirement for annotation despite providing an efficient route to the generation of new hypothesis. By contrast, targeted-omics studies (often incorporating isotope labelling to measure metabolite flux) can be used to measure a specifically predetermined selection of small molecule metabolites, and are often a direct route to interrogating pathways of interest [22]. Multivariate statistics, such as principal component analysis (PCA), an unsupervised statistical method, or a supervised orthogonal partial least squares discriminant analysis (OPLS-DA), can then be used to identify distinctions within groups such as between an experimental and control group, or in the theme of this review, when comparing the drug-resistant and sensitive lines [23].

The aim of this systematic review was to give insight into the current landscape on investigating TKI resistance in NSCLC, with a focus on the metabolic adaptations of TKI-resistant cell populations.

## 2. Materials and Methods

**Figure 2:**
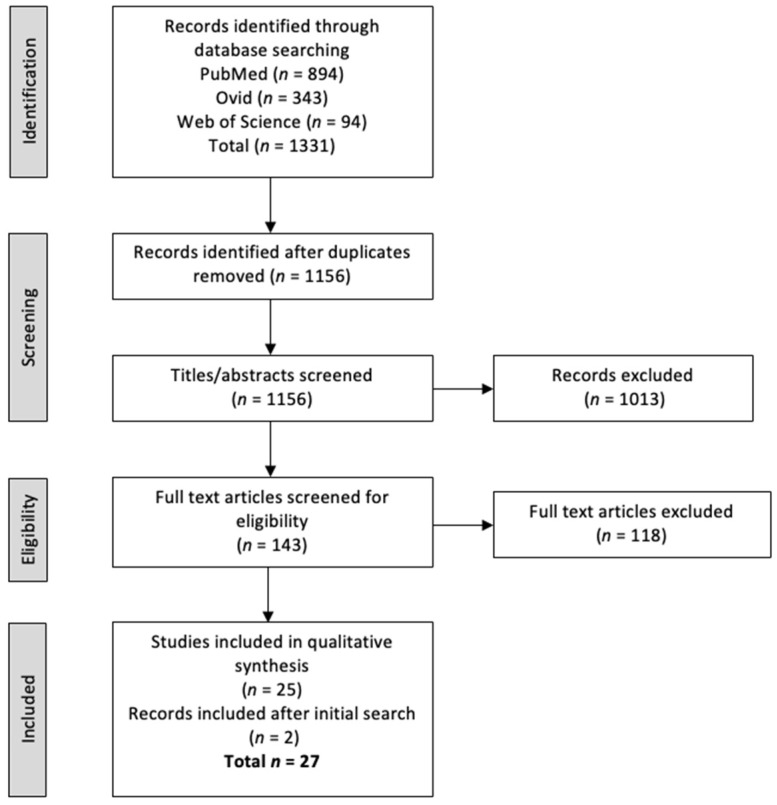
PRISMA flow diagram summarising the review process of all papers. Adapted from Moher et al., 2009 [26].

The review was conducted and written in accordance with the 2020 Preferred Reporting Items for Systematic Reviews and Meta-analyses (PRISMA) guidelines [24]. A literature search of the entirety of PubMed and Ovid (MedLine) and a search of articles only from Web of Science were conducted. The evidence-based PICO (Patient/Population/Problem, Intervention, Comparison and Outcome) model was used to generate relevant search terms to answer the research question “to understand the role of metabolism on the ability for NSCLC cells to acquire resistance to EGFR treatment”. Terms such as “NSCLC”, “metabolomics”, “lipidomics”, “EGFR therapies” and “drug resistance” along with their synonyms were searched (see Supplementary Materials Table S1). Collectively, 1331 studies were retrieved and input to Covidence, a “primary screening and data extraction tool” for those conducting systematic reviews [25], and after 175 duplicate papers were excluded, papers were then excluded based on account of no full text article being available or the studies were not primary literature reports (e.g., books/reviews/letters). Full inclusion and exclusion criteria can be found in the Supplementary Materials (Table S2). Figure 2 depicts the full process of this systematic review based on the PRIMSA guidelines for systematic reviews [26]. Finally, 27 studies were selected for deeper analysis. A summary of these papers can be found in the Supplementary Materials (Table S3).

## 3. Results and Discussion

**Figure 3:**
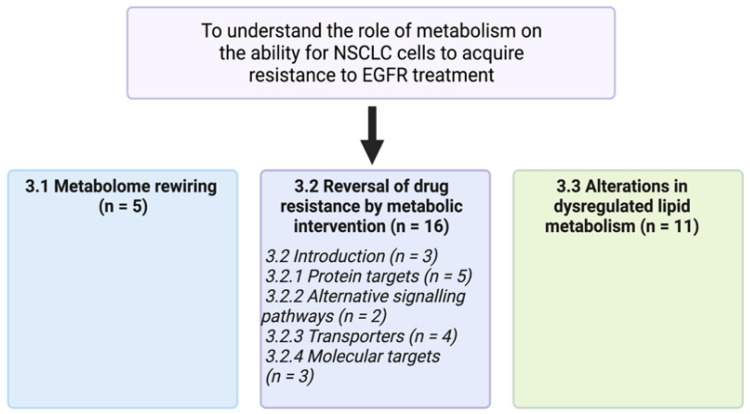
Schematic representation of the main themes of this review. With a total of 27 studies selected for deeper analysis, some papers fall into more than one section.

From the 27 studies selected for comprehensive analysis by review of the full text and all supplementary materials, three main themes were identified: (i) metabolome rewiring, (ii) reversal of drug resistance by metabolic intervention and (iii) alterations in dysregulated lipid metabolism. These are discussed in turn below. Figure 3 is a visual aid representing the number of papers found within each section, with some papers being discussed in more than one section. 

### 3.1. Metabolome Rewiring

The gold standard for measuring the metabolic phenotype of cells is metabolomics; in this context to compare and contrast the metabolite profiles of EFGR-mutant treatment sensitive and treatment resistant NSCLC cells. Our review of the literature highlighted key multiple metabolic pathways altered during the onset of drug resistance.

Using a ^1^H NMR-based metabolomic approach, Li et al., 2016, identified 36 assigned metabolites that differentiated erlotinib-resistant and sensitive cell lines [3]. Many of these were involved in glutathione (GSH) metabolism, amino acid and nucleotide synthesis, and choline metabolism—a precursor for phospholipid synthesis [3]. Interestingly, there was a notable decrease in the intracellular levels of GSH in resistant cell lines. Ma et al., 2020 took a multi-omics approach, utilising data from metabolomics obtained via LC-MS and proteomics to compare osimertinib-resistant and sensitive cells using multivariate statistics [4]. Overall, 54 metabolites were found to have different abundances between resistant and sensitive cells, and these were largely related to amino acid and nucleotide metabolism, implicating these pathways in the development of osimertinib resistance [4].

Using capillary electrophoresis-time of flight MS (CE-TOF-MS), Serizawa et al., 2014, measured the metabolic profile of erlotinib-resistant and sensitive cell lines and determined 18 metabolites that significantly contributed to distinguishing sensitive from resistant cell lines [27]. These included glucose 6-phosphate and other glycolytic intermediates, which were all lower in resistant cells compared to sensitive cells. Furthermore, metabolites relating to glutamine metabolism were higher in resistant cells [27]. This suggests that there was a shift away from glycolysis as the main energy pathway in resistant cells.

Thiagarajan et al., 2016, used transcriptomics and metabolomics, which determined that transforming growth factor beta (TGF-ß) contributed to adaptive drug-escape, a phenomenon where cells are able to acquire new methods of resistance, along with altered metabolic bioenergetics, regulated by mitochondrial function [28]. Mass spectrometry found a global change in energy metabolism when the cells were in their drug-escape phase, with alterations in both the glycolytic and TCA cycle intermediates [28]. There were also significant changes in lipid metabolism, with precursors of fatty acid synthesis elevated in cells after 9 days of erlotinib treatment.

In addition to drug resistance, drug tolerance can also occur [29]. Drug tolerance can be defined as a group of cells which survive the initial exposure to TKIs, and are often the pool of cells in which the majority of drug-resistant cells arise from [30]. Zhang et al., 2019, report that EGFRm drug-resistant cells can emerge from drug tolerant cells [29], and therefore imply that these cells should be considered a therapeutic target to prevent resistance. This study used a targeted metabolomics approach via LC-MS/MS, to identify key mediators in the TCA cycle that were altered, for example increased succinate, which suggested loss of function of succinate dehydrogenase (SDH). SDH is a stabilising agent to the hypoxic inducible factor α (HIF1α), therefore the study concluded that the alteration of the TCA cycle can activate a hypoxic response, enabling drug tolerance to develop [29].

### 3.2. Reversal of Drug Resistance by Metabolic Intervention

**Figure 4:**
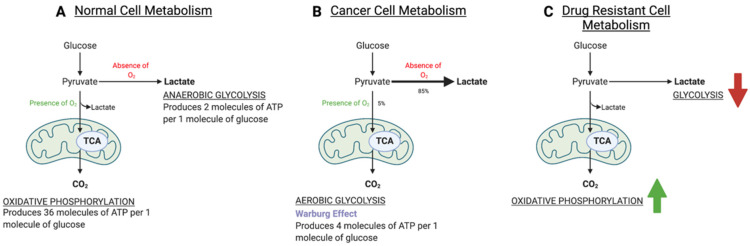
Cellular energy metabolism in (**A**) normal cells in the presence of oxygen, cells produce around 36 ATP molecules per glucose via glycolysis and the TCA cycle. In the absence of oxygen, cells accumulate lactate and produce only 2 ATP molecules. (**B**) Cancer cells both in the presence and absence of oxygen only use glycolysis to produce around 4 ATP molecules, whilst producing more lactate than normal cells in the absence of oxygen. (**C**) Drug-resistant cells favour oxidative phosphorylation to provide energy over aerobic glycolysis.

The Warburg effect, as stated, describes a switch in energy metabolism where cancer cells adapt to gaining their energy supply from aerobic glycolysis, as opposed to oxidative phosphorylation [7,8,9]. Figure 4 shows the pathways of glycolysis and oxidative phosphorylation. Reversing this altered metabolism is, therefore, a way to treat cancer cells.

De Rosa et al., 2015, investigated the effects of EGFR targeting TKIs on glucose metabolism in NSCLC, reversing the Warburg effect [8]. They found that efficient inhibition of EGFR signalling restored oxidative phosphorylation as the primary energy source in NSCLC. This inhibition also regulated aerobic glycolysis via downregulation of hexokinase II (HKII); an enzyme that phosphorylates glucose to glucose-6-phosphate in the initiating step of glycolysis [8] and phospho-pyruvate kinase M2 (p-PKM2); which catalyses the formation of pyruvate from phosphoenolpyruvate (PEP), the final step of glycolysis (See Figure 1 for a schematic of the steps in glycolysis) [31]. Therefore, it is key to understand the mechanisms behind this shift between both energy pathways to prevent or reverse resistance. Kim et al., 2018, found that the mutation within EGFR itself enhanced glycolysis, which was required for EGFR stability [11]. In addition, they determined that depriving resistant cells of glucose inhibited glycolysis, inducing apoptosis.

As a preference for oxidative phosphorylation over glycolysis is a key energy metabolism hallmark of resistant cells, targeting the pathway itself would be one way to revert cells to favouring glycolysis [9]. In a parental EGFRm line, osimertinib supressed glycolysis and favoured oxidative phosphorylation [9], but does not have this effect in resistant lines. By targeting a component of the electron transport chain (ETC); complex I, with an inhibitor metformin, this study found that when used in conjunction with osimertinib, the development of drug resistance was inhibited in vitro [9].

The metabolomic studies in Section 3.1 highlighted that key metabolic pathways are altered in drug resistance including the TCA cycle and glycolysis, with the Warburg effect being an important cancer hallmark. Taking this further, a second core theme of the papers selected in this systematic review was that dysregulated cellular metabolism in TKI-drug resistance can be reversed by an intervention to reinstate drug sensitivity.

#### 3.2.1. Protein Targets

To look at the dysregulation of energy metabolism in TKI-resistant cells, with the intention to re-sensitise cells to treatment and reinstate therapeutic effect, targeting proteins implicated in metabolism was an approach used by several authors [1,32,33,34,35].

5′ adenosine monophosphate-activated protein kinase (AMPK) is a sensory kinase activated by intracellular levels of ATP and AMP [33]. Its downstream targets, such as mTOR and acetyl-CoA carboxylase (ACC) [32], when phosphorylated, redirect metabolism towards increased catabolism [36]. Metabolic stress through direct activation of AMPK by 2-deoxy-D glucose (2DG), which in turn inhibits glycolysis, enhanced cellular sensitivity and therefore the anti-cancer effect of both afatinib and gefitinib in separate studies [32,33].

Pyruvate dehydrogenase (PDHK1) is a protein complex that when activated can inhibit glycolytic activity [1]. To investigate pyruvate metabolism, transcriptomics data from two separate NSCLC cohorts was interrogated and found increased expression of PDHK1. Upon inhibition with dichloroacetate (DCA) in combination with an EGFR inhibitor, metabolism was rewired to favour pyruvate oxidation whilst reducing lactate production, therefore inhibiting glycolysis and increasing the therapeutic effect of the EGFR inhibitor [1].

Targeting glutamine metabolism, a key nutrient for cancer cells, can also be a potential strategy to overcome drug resistance in NSCLC [34]. A compound that targeted glutaminase C (GAC) in combination with erlotinib downregulated both glutamine and glucose metabolism in erlotinib-resistant cells [34].

#### 3.2.2. Alternative Signalling Pathways

A further approach to reinstate sensitivity to TKIs and downregulate resistant cells’ requirement for glycolysis is by targeting alternative signalling pathways.

Ye et al., 2017, targeted AKT expression in resistant cells in combination with inhibiting glucose metabolism [37]. AKT and autophagy were found to be more activated in resistant cells than in the parental line. The study found that an increased uptake of glucose via GLUT1, a major glucose transporter, was a feature of resistant cells, and removing glucose would reverse resistance. However, drug sensitivity did not increase until they combined this with suppressing AKT phosphorylation via an inhibitor MK2206 [37].

Cellular metabolism is known to change when cancer cells are under hypoxic conditions, due to the lack of oxygen to the cells. This can result in drug resistance for those drugs which require oxygen to be cytotoxic, or of interest in this review, by the alteration of metabolism [38]. In hypoxic conditions, HIFs are activated and can induce a multidrug efflux transporter, p-glycoprotein, and therefore convey drug resistance [4]. Ma et al., 2020, found that expression of upstream proteins of HIF1α were significantly increased in osimertinib-resistant cells. Bypass signalling pathways, such as the PI3K/Akt pathway, were also found to be enriched in osimertinib-resistant cells [4,37].

#### 3.2.3. Transporters

Transporters play key roles in determining cellular energy fate. An upregulation of a key transporter involved in glycolysis for example, such as GLUT1, would result in more glucose being transported into the cell [37], and if you inhibit this with an inhibitor such as dehydroascorbate (DHA), which is oxidized vitamin C and taken up by GLUT1, glycolysis is inhibited [39]. Another study found that GLUT1 expression, and therefore glucose uptake, were more prominent in gefitinib-resistant cells, and once genetic and pharmacological inhibition of GLUT1 was established, resistant cells were re-sensitised to therapy [40]. One study found that monocarboxylate transporter 1 (MCT-1), a transporter that secretes lactate into extracellular space, was upregulated in TKI-resistant cells compared to sensitive cells [41].

#### 3.2.4. Molecular Targets

Bach et al., 2018, investigated bone morphogenetic proteins (BMPs), which belong to the transforming growth factor ß (TGF-ß) family and control many processes including cell differentiation and tumour growth [42]. Activation of BMPs has been known to confer resistance to EGFR-TKIs and to test this Bach et al., 2018, used gefitinib-resistant cells to determine the gene expression of these cells compared to parental sensitive cells [42]. BMP4 plays a role in energy metabolism through regulation of acyl-CoA synthetase long chain family member number 4 (ACSL4), which is key in lipid metabolism and fatty acid oxidation, as it catalyses the conversion of polyunsaturated fatty acids (PUFAs) to acyl-CoAs [42]. TGF-ß was also found to aide adaptive drug-escape through transcriptomics and metabolomics, with cells at this point able to exhibit adaptive metabolic bioenergetics, once again linking back to mitochondrial function [28].

mTORC2 was found to play a role in metabolic reprogramming in erlotinib-resistant cells, as these cells had more metabolic flexibility when compared to sensitive cells, measured via spare respiratory capacity (SRC). SRC is a measure of the extra mitochondrial availability, i.e., the ability for increased ATP production via oxidative phosphorylation, and is an indicator of metabolic reprogramming [43]. Knockdown of an mTORC2 component, Rictor, increased SRC in resistant cells. This, therefore, implies that mTORC2 mediated the metabolic reprogramming of resistant cells, as resistant cells before treatment intervention have lower SRC and therefore lower mitochondrial activity [43].

### 3.3. Alterations in Dysregulated Lipid Metabolism

Lipid metabolism is tightly linked to glycolysis and oxidative phosphorylation, see Figure 1 [13,44]. Lipid metabolism has been found to be dysregulated in TKI-resistant cells compared to TKI-sensitive cells. In particular, de novo lipogenesis is favoured by resistant cells as opposed to utilising endogenous lipids [19]. FASN is a key enzyme required for de novo lipogenesis and is frequently upregulated in cancer, whilst SREBPs are master transcription factors that regulate lipid and cholesterol metabolism [45,46,47]. Ali et al., 2018 demonstrated a novel link between EGFR signalling and FASN expression in resistant cells. EGFR signalling knockdown decreased levels of both FASN and its activator SREBP1, demonstrating that blocking lipid synthesis can be of therapeutic benefit to resistant cells [45]. This was then confirmed with pharmacological inhibition of FASN using orlistat, which had a cytotoxic effect on gefitinib-resistant cells [45].

Chen et al., 2021, also determined a link between SREBP1 and EGFRm response to osimertinib, which found that the mature form of SREBP1 (mSREBP1) was degraded in the presence of osimertinib, suppressing lipogenesis. However, once these cells acquired resistance to osimertinib, the ability to suppress lipogenesis was lost, and mSREBP1 levels began to increase once more [20]. This study used untargeted lipidomic analysis on osimertinib-treated cells compared to vehicle to determine how treatment changed the lipidomic profile of cells. This identified 148 lipid metabolites, with 50 of these including TAGs, DAGs, ceramides (CERs), PEs, sphingomyelins (SMs) and PUFA PEs showing a significant decrease in abundance in osimertinib treated cells, highlighting how drug treatment can change the lipidomic phenotype of a cell [20].

Xu et al., 2021, used lipidomics to show that gefitinib treatment altered the ratio of saturated and unsaturated phospholipids in resistant and sensitive cell lines, highlighting that prolonged SREBP1 activation is key for de novo lipogenesis, and resistant cells do in fact rely upon this mechanism [19]. Another investigation in SREBP1 signalling by Li et al., 2016, found that intervening with MARVELD1, a SREBP binding partner, inhibits lipogenesis and improves therapeutic effects of TKIs [47]. As mentioned above, when AMPK is activated, it acts upon ACC, which is vital for the de novo fatty acid biosynthesis pathway. Its activation incurred metabolic stress and inhibited glycolysis, which enhanced TKI therapeutic effect [32,33].

Cholesterol is an essential cell membrane lipid and plays an integral role in maintaining cell function and integrity [48]. Cholesterol exists in pockets of the plasma membrane, known as lipid rafts [48]. In gefitinib-resistant cells, cholesterol levels within these lipid rafts were higher than in gefitinib-sensitive cells. This knowledge prompted Chen et al., 2018, to determine if there was a link between intracellular cholesterol levels and resistance to EGFR-TKIs [48]. They found that cholesterol inhibited binding of gefitinib to EGFR, resulting in resistance. Overall, depletion of cholesterol levels restored drug sensitivity in these resistant cell lines [48]. An alternative strategy to target high cholesterol levels in resistant cells is to target the key mediator of cholesterol uptake; the low-density lipoprotein receptor (LDLR), which is upregulated via EGFR activation through SREBP1 [49]. Despite this study focusing more on overall therapeutic benefit of TKIs as opposed to resistance, Luo et al., 2021, highlighted another potential way to reduce cholesterol levels.

SCD1 is a further key enzyme in lipogenesis, responsible for synthesizing saturated fatty acids (SFAs) into monounsaturated fatty acids (MUFAs) [50]. These can then be incorporated into neutral lipids that are stored in lipid droplets (LDs), which have a high expression in cancer cells [50]. Therefore, Huang et al., 2019, investigated the link between SCD1 mediated lipogenesis and TKI resistance. Intracellular LDs were found to be of a higher proportion in resistant cells, along with expression of SCD1 and its enzymatic product oleic acid. The resistance was overcome by combined pharmacological inhibition of SCD1 with 20(S)-protopanaxatriol and EGFR-TKI [50].

Jung et al., 2015, set out to investigate the phospholipid composition of extracellular vesicles (EVs) that tumour cells secrete [51]. Using matrix-assisted laser desorption/ionization (MALDI) MS to look at lipid profiles of resistant and sensitive cells, this study found that there were 67 phospholipids, including PCs, LysoPCs, SMs, PGs, PIs and LysoPIs, that had varying abundances in resistant vs. sensitive cells [51]. This result highlights once again that resistant and sensitive cells possess unique lipidomic phenotypes, suggesting a novel way to reinstate therapeutic effect.

## 4. Conclusions

The investigation into cellular metabolism dysregulation in EGFR-TKI resistant cells compared to those cells that are still sensitive to drug treatment, can elucidate several mechanisms within key pathways that may be targeted to prevent or reverse resistance.

The Warburg effect is a well characterised hallmark of cancer and alludes to the switch seen in cancer cells in which they favour aerobic glycolysis over oxidative phosphorylation, even in oxygenated conditions [52]. However, the reverse is true of drug-resistant cell populations [9]. More recently, perturbed lipid metabolism has been described in cancer cells and drug-resistant cells. In particular, de novo lipogenesis has been found to be upregulated in drug-resistant cells compared to sensitive cells [20].

This systematic review has highlighted the importance of investigating metabolomic and lipidomic remodelling, and the pathways that are altered allowing resistant cells to dysregulate cellular metabolism. In the papers summarised here, the studies use both metabolomics and lipidomics to investigate the phenotype of resistant cells, to understand the changes observed from gene expression to metabolite abundance.

By elucidating the metabolome and lipidome changes found within resistant cells and comparing these to sensitive cells, specific metabolic pathways can be targeted alongside EGFR-TKI treatment to allow continued therapeutic benefit.

## Data Availability

The data set (27 papers) analysed in this study is available upon request from the corresponding author.

## Supplementary Materials

The following supporting information can be downloaded at: https://www.mdpi.com/article/10.3390/metabo12070644/s1, Table S1: Search terms used in the literature search across all databases; Table S2: Inclusion and exclusion criteria used for literature selection; Table S3: A brief summary of all 27 papers included in the full analysis of this systematic review.
