# Supplementary material for: Dysregulated Metabolism in EGFR-TKI Drug Resistant Non-Small-Cell Lung Cancer: A Systematic Review
Source: Metabolites. 2022 Jul 14;12(7):644. doi: 10.3390/metabo12070644 (PMC9316206; doi:10.3390/metabo12070644)
Supplement: Supplementary file 1 [file metabolites-12-00644-s001.zip › metabolites-1806486-supplementary.pdf]

Dysregulated Metabolism in EGFR-TKI Drug Resistant Non-Small-Cell Lung Cancer: A Systematic Review

Julia Babuta, Zoe Hall and Toby Athersuch

Supplementary Materials:

Table S1. Search terms used in the literature search across all databases.

Entry	Keyword
1	NSCLC.mp
2	Non small cell lung cancer.mp
3	Non-small-cell lung cancer.mp
4	**1 or 2 or 3**
5	Osimertinib.mp
6	Tagrisso.mp
7	Erlotinib.mp
8	Tarceva.mp
9	Gefitinib.mp
10	Iressa.mp
11	3rd line.mp
12	3rd generation.mp
13	Tyrosine kinase inhibitor.mp
14	EGFR therapy.mp
15	EGFR-targeted therapies.mp
16	EGFR-TKI.mp
17	Epidermal growth factor receptor tyrosine kinase inhibitors.mp
18	**5 or 6 or 7 or 8 or 9 or 10 or 11 or 12 or 13 or 14 or 15 or 16 or 17**
19	Epidermal growth factor receptor.mp
20	EGFR.mp
21	T790M.mp
22	EGFRm.mp
23	**19 or 20 or 21 or 22**
24	metabolomics.mp
25	metabolite.mp
26	metabolome.mp
27	metabolic profiling.mp
28	metabolic biomarker.mp
29	metabolism.mp
30	metabolic profile.mp
31	metabonomics.mp
32	metabolic phenotyping.mp

33	small molecule metabolites.mp
34	lipidomics.mp
35	**24 or 25 or 26 or 27 or 28 or 29 or 30 or 31 or 32 or 33 or 34**
36	drug resistance.mp
37	resistance.mp
38	36 or 37
39	4 and 18 and 23 and 35 and 38

Table S2. Inclusion and exclusion criteria used for literature selection.

Inclusion Criteria	Exclusion Criteria
English full text articles	Proteomic studies
Metabolites	No full text
Metabolomic techniques (e.g., LC-MS, GC-MS, NMR spectroscopy)	Non-EGFR treatments/pathways
EGFR therapies	Books, reviews, letters, conference abstracts
Original data/results	New treatments to overcome TKI resistance
TKI resistance	RNAseq
Genes/pathways linked to metabolism	Other cancers with EGFR treatment
Metabolic pathways (glycolysis) etc.	

Table S3: A brief summary of all 27 papers included in the analysis of this systematic review.

Study	Drug Treatment	Cell Lines	Methods	Statistical Analysis	Summary of Findings
Dyrstad et al., 2021[1]	Erlotinib	HCC827, H1975 (erlotinib resistant and sensitive)	1) Seahorse XF assay to measure OCR<br>2) CO2 trapping	ANOVA and unpaired two-tailed t-test and $p < 0.05$ considered statistically significant	Targeted glycolysis to prevent TKI resistance. Inhibited PDHK activity as this is increased in resistant cells. Redirecting metabolism to restore therapeutic effect of EGFR-TKI.
Li H et al., 2016[3]	Erlotinib	PC9, H3255, H1975	1) Metabolomics:<br>^{1}H-NMR<br>2) GSH colorimetric assay	OPLS-DA	Erlotinib resistant cells were re-sensitised to TKI when GSH was increased. 36 differential metabolites, 14 involved in GSH, amino acids, nucleotides and choline metabolism correlated with resistance.
Ma et al., 2020[4]	Osimertinib	H1975 (osimertinib resistant and sensitive)	1) Metabolomics and proteomics:<br>LC-MS – Linear ion trap-Orbitrap	PCA, OPLS-DA,<br>two-tailed t-test and $p < 0.05$ considered statistically significant	54 differential metabolites found all related to amino acid metabolism. Increased reliance on oxidative phosphorylation, and decreased glycolysis in resistant cells. Increase in PI3k/Akt signaling and HIF1α in resistance cells and a decrease in arginine/proline metabolism.
DeRosa et al., 2015[8]	Erlotinib	H1975, HCC827, H1993	1) F-FDG glucose uptake and consumption<br>2) Lactate secretion<br>3) Intracellular ATP levels<br>4) Seahorse XF assay to measure OCR and ECAR	Unpaired and paired t-test and $p < 0.05$ considered statistically significant	EGFR-TKIs reactivated oxidative phosphorylation, this shift was mediated by PI3K/Akt and RAS/MAPK.
Martin et al., 2016[9]	Osimertinib	PC9, H1975 (osimertinib resistant and sensitive)	1) Lactate assay<br>2) Seahorse XF assay to measure OCR	Two-tailed t-test and $p < 0.05$ considered statistically significant	Osimertinib suppressed glycolysis in sensitive lines. oxidative phosphorylation inhibitor delayed or prevented drug resistance. Osimertinib treatment induced oxidative phosphorylation.
Kim et al., 2018[11]	Gefitinib<br>Erlotinib	A549, H1299, HCC827, H1975, PC9 (PC9 gefitinib and erlotinib resistant and sensitive)	1) Metabolomics:<br>LC-MS<br>2) Glucose consumption/Lactate production assay<br>3) Seahorse XF assay to measure OCR and ECAR<br>4) ROS quantification<br>5) Intracellular ATP quantification	t-test and p-value < 0.05 considered statistically significant	EGFR-TKI can overcome resistance via inhibition of glucose metabolism or JNK activation.
Xu et al., 2021[19]	Gefitinib	H1975, PC9 (gefitinib resistant and sensitive)	1) Lipidomics:<br>ESI-MS/MS on AB SCIEX Triple TOFTM 5600+<br>2) Intracellular measurements of fatty acid, cholesterol and triglyceride levels	Two-tailed t-test and $p < 0.05$ considered statistically significant	Gefitinib induced downregulation of SREBP1 in sensitive cells only. Gefitinib altered the ration of saturated and unsaturated phospholipids in sensitive vs resistant cells. Inhibition of SREBP1 resensitised cells to gefitinib.
Chen et al., 2021[20]	Osimertinib	PC9, HCC827	1) Lipidomics:<br>LC-MS		Lipogenesis was suppressed by osimertinib treatment as it degraded mSREBP1. Osimertinib treated cells showed a

				Two-sided unpaired Student's t-test and $p < 0.05$ considered statistically significant	decrease in abundance of 50 lipid metabolites, highlighting that drug treatment can change the lipid profile of cells.
Serizawa et al., 2014[27]	Erlotinib	PC9 (erlotinib resistant and sensitive)	1) Metabolomics: CE-TOF-MS	Welch's t-test and p-value < 0.01 considered statistically significant OPLS-DA	Glutamine metabolism was enhanced in erlotinib resistant cells compared to glycolysis. Resistance occurred via constitutive activation of CRKL/PI3K/Akt. 5 genes involved in glutamine/glutathione synthesis had significant gains in copy number in resistant cells. 18/24 metabolites contributed to erlotinib resistance.
Thiagarajan et al., 2016[28]	Erlotinib	HCC827, H1975	1) Affymetrix Array 2) Glutamine deprivation 3) Metabolomics	PCA	TGFβ2 upregulation increased EMT-ness and remarkable adaptive transcriptome-metabolome bioenergetic alterations correlated with early-onset drug escape of EGFRm NSCLC. Global reduction in glucose metabolism, TCA cycle activity and reduction of synthesis in fatty acids and membrane lipids.
Zhang et al., 2019[29]	Osimertinib Gefitinib	H358, H460, A549, H1299, H69, H1650, H1975, HCC827 (osimertinib and gefitinib resistant and sensitive), PC9 (osimertinib and gefitinib resistant and sensitive), H3255	1) Metabolomics: HPLC-MS/QTRAP Triple-Quadrupole	t-test and p-value < 0.05 considered statistically significant	Increased levels of miR-147b created a drug tolerance for EGFR inhibition. This disrupted the TCA cycle and activation of hypoxic responses irrespective of oxygen levels.
Chen et al., 2017[32]	Gefitinib	H1975, H820, H1299	1) Glycolysis stress test 2) Cholesterol staining/quantity assay	One-way ANOVA and Dunnett's test, $^{*}p < 0.05$, $^{**}p < 0.01$, $^{***}p < 0.001$ considered statistically significant	AMPK regulated metabolic homeostasis through inducing metabolic changes to balance energy consumption and production intracellularly. AMPK inhibitor shows cytotoxicity towards both gefitinib sensitive and resistant cells.
Kim et al., 2013[33]	Afatinib	H1975, PC9 (afatinib resistant and sensitive)	1) Lactate production assay 2) Orimetric assay for intracellular ATP quantification	t-test and p-value < 0.05 considered statistically significant	Inhibiting glycolysis by 2DG improves TKI efficacy in all cell lines used. Downregulation of MCL1 via alteration of AMPK/mTOR signaling improved afatinib efficacy via inhibition of glycolysis.
Xie et al., 2016[34]	Erlotinib	HCC827 (erlotinib resistant and sensitive)	1) Metabolomics: ^{1}H-NMR spectroscopy 2) Glutaminase activity assay	PCA One way ANOVA with p-value < 0.05 considered statistically significant	Inhibited glutaminase which inhibited cell proliferation in resistant cells and in combination downregulated glutamine and glycolysis metabolism, implying that inhibition with compound 968 and TKI inhibited whole cellular metabolism.
Yang et al., 2018[35]	Erlotinib	H1975, H1650	1) Seahorse XF assay to measure OCR 2) Detection of intracellular ATP 3) Metabolomics: LC-MS	ANOVA, t-test and p-value < 0.05 considered statistically significant	PDK1 regulates glycolysis. Inhibiting this enhanced TKI effect in hypoxic conditions and improved oxidative phosphorylation. This increased the anti-cancer efficacy of erlotinib.
Ye et al., 2017[37]	Erlotinib	HCC827 (erlotinib resistant and sensitive)	1) Seahorse XF assay to measure OCR and ECAR 2) ROS, GSH and GSSG measurement 3) Metabolomics:		Mitochondrial dysfunction plays a role in the erlotinib resistance as mitochondria rely on glycolysis. Blocking

			UPLC-TOF for metabolites 4) Lactic acid and glucose measurements	ANOVA and p-value < 0.05 considered statistically significant	autophagy/AKT and causing glucose deprivation re-sensitised the cells to TKI.
Ye et al., 2019[39]	Erlotinib	HCC827 (erlotinib resistant and sensitive)	1) Seahorse XF assay to measure OCR and ECAR 2) Metabolomics: UPLC-TOF	ANOVA with p-value < 0.05 considered statistically significant	Compared the sensitive and resistant cell lines. Erlotinib resistant cells switched to higher glycolysis as opposed to oxidative phosphorylation like the other findings. Used Vitamin C to inhibit this glycolysis and prevent drug resistance.
Suzuki et al., 2018[40]	Gefitinib Erlotinib	A549, H1299, PC9, HCC827	1) Glucose uptake assay	Two-tailed t-test (with Bonferroni correction) and p < 0.05 considered statistically significant	GLUT1 mediated glucose metabolism led to gefitinib resistance. Targeting GLUT1 overexpression returned sensitivity to gefitinib.
Huang et al., 2020[41]	Gefitinib	PE089 (gefitinib resistant and sensitive), PF001 (gefitinib sensitive), PF002 (gefitinib resistant)	1) Mitochondrial extraction 2) Seahorse XF assay to measure OCR and mitochondrial complex activity	t-test and p-value < 0.05 considered statistically significant	Mitochondrial translocation allowed for EGFR-TKI resistant cells to switch from glycolysis to oxidative phosphorylation. EGFR translocation to the mitochondria shifted metabolism and increased MCT1, LDHB and PDHA1. The resistant cells responded to an MCT1 inhibitor.
Bach et al., 2018[42]	Erlotinib Gefitinib	H1993 (gefitinib/erlotinib resistance)	1) Affymetrix Array 2) Metabolomics: 1H-NMR spectroscopy	t-test and *p < 0.05, **p < 0.01, ***p < 0.001 considered statistically significant	Association between gefitinib resistant NSCLC cells and BMP4 overexpression. BMP4 interacts with lipid metabolism via ACSL4 and p53 signaling pathway.
Chiang et al., 2018[43]	Erlotinib	PC9, H3255 (erlotinib resistant and sensitive) HCC4011 (MET amplified)	1) Agilent Seahorse XF metabolic assay	Two-tailed t-test and *p < 0.05, **p < 0.01, ***p < 0.001 considered statistically significant	mTORC2 metabolically reprogrammed erlotinib resistant cells therefore mTOR plays a role in glucose metabolism regulation.
Ali et al., 2018[45]	Gefitinib	EGFRm – PC9, HCC4006, H1650, H820 and H1975 EGFRwt – H1703 Immortalized non-transformed lung NL20	1) Free fatty acid measurement	t-test and p < 0.05 considered statistically significant	EGFR signaling relies upon EGFR palmitoylation. FASN regulation by EGFRm mediated TKI resistance. Targeting FASN with Orlistat halts EGFRm signaling. Fatty acid metabolic pathway is upregulated in EGFRm NSCLC cells with acquired resistance to gefitinib. Knockdown of EGFR signaling decreased levels of both FASN and its activator SREBP1, implying that blocking the fatty acid metabolism can therapeutically benefit resistant cells.
Li et al., 2016[47]	Gefitinib	PC9, A549	1) Lipidomics: GC-MS 2) Triglyceride and cholesterol assay	t-test and *p < 0.05, **p < 0.01, ***p < 0.001 considered statistically significant	Gefitinib sensitivity was enhanced when knockdown of MARVELD1 occurred (usually binds to SREBP1 to stimulate lipid metabolism). Reduced proportion of unsaturated fatty acids (by SREBP) in cell membrane resulted in decreased cell membrane fluidity.
Chen et al., 2018[48]	Gefitinib	A549, H1975, H1650, PC9 (gefitinib resistant and sensitive)	1) Lipid raft isolation 2) Amplex red cholesterol assay	Two-tailed t-test and p < 0.05 considered statistically significant	Cholesterol levels were higher in gefitinib resistant cells compared to sensitive cells. Lovastatin reduced cholesterol levels to inhibit tumor growth in gefitinib resistant cells.

Luo et al., 2021[49]	Gefitinib Osimertinib	H1993, A549, PC9, HCC827 and H1975	1) Immunoblotting 2) MTT 3) siRNA	Two-tailed t-test and p < 0.05 considered statistically significant	EGFR signaling upregulated LDLR expression via SREBP1. EGFRm count on lipids for survival. Combination of a statin and TKI could improve treatment.
Huang et al., 2019[50]	Gefitinib	HCC827, PC9, H1975 (gefitinib resistant and sensitive)	1) BODIPY 493/503 staining for LD 2) Treatment with OA to simulate high lipid environment	t-test followed by one-way ANOVA and Tukey's post-test. p-value < 0.05 considered statistically significant	Main link between lipid metabolic reprogramming and TKI resistance. Hypothesized that LD metabolized into small molecule phospholipids and fatty acids to maintain the survival of resistant cells.
Jung et al., 2015[51]	Gefitinib	PC9 (gefitinib resistant and sensitive)	1) Lipidomics: Lipid MALDI-MS and Triple Quadrupole LC-MS	PCA	35 differentially expressed lipids between resistance and sensitive cells. EV's contain lipid signatures including phospholipid changes that related to drug resistance (67 differentially expressed phospholipids identified).

Abbreviations: AMPK; AMP-activated protein kinase, BMP4; bone morphogenic protein 4, CE-TOF-MS; capillary electrophoresis-time of flight-MS, ECAR; extracellular acidification rate, EGFR-TKI; epidermal growth factor receptor-tyrosine kinase inhibitor, ESI; electron spray ionisation, FASN; fatty acid synthase, F-FDG; fluorodeoxyglucose, GSH; glutathione, HPLC-MS; high performance liquid chromatography MS, LC-MS; liquid chromatography-mass spectrometry, LDLR; low density lipoprotein receptor, LD; lipid droplet, MTT; 3-(4,5-dimethyl-2-thiazolyl)-2,5-diphenyltetrazolium bromide, NMR; nuclear magnetic resonance, OCR; oxygen consumption rate, PDHK; pyruvate dehydrogenase kinase, ROS; reactive oxygen species, SREBP1; sterol regulatory binding protein 1, mSREBP1; matureSREBP1.
